# The Extent, Range, and Nature of Quantitative Nutrition Research Engaging with Intersectional Inequalities: A Systematic Scoping Review

**DOI:** 10.1016/j.advnut.2024.100237

**Published:** 2024-05-04

**Authors:** Emily Fivian, Helen Harris-Fry, Claudia Offner, Michele Zaman, Bhavani Shankar, Elizabeth Allen, Suneetha Kadiyala

**Affiliations:** 1 Department of Population Health, Faculty of Epidemiology and Population Health, London School of Hygiene and Tropical Medicine, London, United Kingdom; 2 Department of Medicine, Queen’s University, Ontario, Canada; 3 Department of Geography, The University of Sheffield, Sheffield, United Kingdom; 4 Department of Medical Statistics, Faculty of Epidemiology and Population Health, London School of Hygiene and Tropical Medicine, London, United Kingdom

**Keywords:** intersecting inequalities, intersectionality, nutrition, diets, food security, scoping review, malnutrition, social determinants of health, inequalities in nutrition, quantitative research methods

## Abstract

Addressing malnutrition for all requires understanding inequalities in nutrition outcomes and how they intersect. Intersectionality is increasingly used as a theoretical tool for understanding how social characteristics intersect to shape inequalities in health outcomes. However, little is known about the extent, range, and nature of quantitative nutrition research engaging with intersectional inequalities. This systematic scoping review aimed to address this gap. Between 15 May 2021 and 15 May 2022, we searched 8 databases. Studies eligible for inclusion used any quantitative research methodology and aimed to investigate how social characteristics intersect to influence nutrition outcomes. In total, 55 studies were included, with 85% published since 2015. Studies spanned populations in 14 countries but were concentrated in the United States (*n* = 35) and India (*n* = 7), with just 1 in a low-income country (Mozambique). Race or ethnicity and gender were most commonly intersected (*n* = 20), and body mass index and overweight and/or obesity were the most common outcomes. No studies investigated indicators of infant and young child feeding or micronutrient status. Study designs were mostly cross-sectional (80%); no mixed-method or interventional research was identified. Regression with interaction terms was the most prevalent method (*n* = 26); 2 of 15 studies using nonlinear models took extra steps to assess interaction on the additive scale, as recommended for understanding intersectionality and assessing public health impacts. Nine studies investigated mechanisms that may explain why intersectional inequalities in nutrition outcomes exist, but intervention-relevant interpretations were mostly limited. We conclude that quantitative nutrition research engaging with intersectionality is gaining traction but is mostly limited to the United States and India. Future research must consider the intersectionality of a wider spectrum of public health nutrition challenges across diverse settings and use more robust and mixed-method research to identify specific interventions for addressing intersectional inequalities in nutrition outcomes. Data systems in nutrition must improve to facilitate this.

This review was registered in PROSPERO as CRD42021253339.

**Statement of Significance**

To our knowledge, this systematic scoping review is the first to describe the extent, nature, and range of quantitative nutrition research engaging with intersectional inequalities, outline gaps in the evidence and implementation of its approach, and highlight future research needs. These findings inform how future research can advance this research agenda in a policy-relevant way so that interventions can be better designed to identify, reach, and benefit the most nutritionally vulnerable individuals.

## Introduction

Good nutrition is a fundamental human right, yet stark differences in nutrition outcomes persist between and within countries, denying many individuals this right. The Sustainable Development Goals (SDGs) demonstrate that ending all forms of malnutrition (SDG2) and reducing inequalities (SDG10) are global priorities [1]. Embedded within these goals is the commitment to leave no one behind [2]. However, no country is on course to meet nutrition targets, as within-country inequalities in nutrition outcomes hinder progress. It is now recognized that addressing these nutrition inequalities requires confronting the systematic power imbalances and social hierarchies driving them [3]. Attention to nutrition inequalities has gained momentum in recent years, both in academic literature [[4], [5], [6]] and across the nutrition policy agenda [7].

However, this work often overlooks how different aspects of marginalization interconnect. For example, although the 2020 Global Nutrition Report [7] and 2021 Lancet Series on Maternal and Child Undernutrition [4] documented wide inequalities in the prevalence of malnutrition along singular social dimensions such as wealth, education, and gender, these estimates conceal the burden of malnutrition among those who experience multiple and interconnected forms of marginalization [8]. Consequently, to accelerate reductions in malnutrition and inequalities in these outcomes, we require a better understanding of which inequalities matter most, how they intersect, and what the consequences for nutrition are.

Intersectionality is a framework that recognizes how an individual’s multiple social characteristics, such as their gender, ethnicity, or class, intersect within complex systems of interlocking power and oppression and shape unequal opportunity for health and wellbeing [9,10]. The relevance of intersectionality to public health [11] and potential for deepening our understanding of and addressing inequalities in nutrition outcomes is increasingly recognized [12,13].

Premises of intersectionality that have been highlighted as important to public health are as follows [14]: *1*) social identities are multiple and mutually constituted; *2*) the starting focus should be on people of multiple historically oppressed and disadvantaged groups; and *3*) intersecting individual social identities (e.g., class and gender) interact with complex macrolevel processes (e.g., classism and sexism) to produce unequal health risks. As such, intersectionality focuses on who experiences different outcomes and also the mechanisms that explain why these differences occur [15]. This is critical to inform the design of public health policies and interventions that reach and benefit those with the greatest need [16].

In the past, intersectionality was mainly applied within qualitative research [[17], [18], [19], [20]], with quantitative research gaining traction more recently. Although qualitative data are well-suited to capturing complex experiences of interlocking disadvantages, quantitative intersectionality could provide insights into the distribution of malnutrition across a broad range of intersecting social characteristics and provide evidence generalizable to large populations [21,22]. A previous review characterized and evaluated how intersectionality was integrated into quantitative research [23]. The review found that most articles studied race, ethnicity, and gender, and 50% studied a health-related outcome. The authors concluded that due to poor engagement with how intersectional social characteristics reflect social power and how theory connects with methods and interpretation, the core theoretical tenets of intersectionality are often diluted in quantitative research [23].

Furthermore, although various statistical methods are being applied to investigate intersectionality within quantitative research [16,22,[24], [25], [26], [27]], reviews have found that most methods are misapplied or misinterpreted and lack adequate explanation [23,28]. Although there is no consensus on which method is most suited to investigate intersectionality, there is consensus that methods should allow social characteristics to combine nonadditively [22]. This is because intersectionality recognizes that an individual’s multiple social characteristics often pertain to related social processes [29]. For example, if classism and sexism cause nutritional vulnerability through interrelated processes, then class and gender will combine in a nonadditive way. However, if gender had no social meaning and was merely a biological facet that caused sex differences in nutrition outcomes, then the additive assumption may hold because the etiology of the outcome concerns 2 unrelated processes [30]. A common way to model this is using linear regression with interaction terms for the social characteristics [23,28]. However, when outcomes are modeled using nonlinear models (e.g., logistic or Poisson), the default interaction is on the multiplicative (not additive) scale. Extra steps can be taken to compute interactions on the additive scale from nonlinear models [31]; these measures are considered more appropriate for intersectionality analyses and for assessing the public health impacts of the interaction [32].

Currently, little is known about the extent, range, and nature of the quantitative evidence on intersectional inequalities in nutrition. To fill this gap and identify future research priorities, we conducted a systematic scoping review with the following research questions: *1*) where and with whom has the research taken place? *2*) which nutrition outcomes, social characteristics, and intersectional relationships have been studied? *3*) which study designs have been implemented? and *4*) what methods have been used to describe and explain intersectional inequalities in nutrition outcomes, and what are the key findings?

## Methods

### Systematic scoping review

As intersectional social characteristics are context-specific and our review spans multiple contexts, we used a systematic scoping review approach. This is a suitable strategy for synthesizing literature that is not amenable to a narrower systematic review [33]. We followed the PRISMA-Extension for Scoping Reviews checklist and guidelines [34] and preregistered our protocol [35].

We note some deviations from our protocol [35]: given the recent publication of systematic reviews of the statistical methods used in quantitative intersectionality literature [23,28], we shifted our review to describe the extent, nature, and characteristics of the nutrition evidence more broadly, with less focus on appraising the strengths and limitations of the statistical methods.

### Data collection

#### Search strategy

After several exploratory searches and reviewing relevant articles, we developed a search strategy using Ovid Medline. Between 15 May 2021 and 15 May 2022, we searched Medline, Embase, Web of Science, Global Health, Ebsco Discovery (limited to AGRIS, Repec, and World Bank e-library), Scopus, Econlit, and ProQuest Dissertations and Theses (limited to dissertations and theses and working papers). Search terms included: “nutrition outcome” and “intersectionality” or [“intersecting” and (“inequality” or “inequity” or “social category”)]. The search strategy for Ovid Medline is provided as an example (Supplemental Methods 1). There was no date restriction, and studies not written in English were ineligible. We also tracked citations of, and references used in, included studies.

#### Inclusion criteria

Studies were eligible for inclusion if they met all of the following criteria: *1*) used any quantitative methodology; *2*) had a nutrition outcome measured at the household or individual level (such as anthropometric measurements, micronutrient status, dietary intake, infant and young child feeding practices, and indicators of food security); *3*) assessed nutrition outcomes based on at least 2 social characteristics pertaining to the individual or household (examples include gender, race, geography/place of residence, and economic status). Articles using community-level characteristics (for example, community-level measures of deprivation or fast food outlet density) to indicate intersectional groups were excluded but included if community-level characteristics were used to indicate potential drivers or mechanisms that may explain intersectional inequalities in nutrition outcomes; and *4*) fell into one of the following 3 categories that indicate engagement with intersectionality:

1. *Framework:* Studies that explicitly referenced the intersectionality framework.
2. *Approach:* Studies that stated taking an “intersectional approach” or used other similar terminology, such as “intersectional analysis” or “intersectional inequalities,” but did not refer directly to the intersectionality framework.
3. *Aim:* Studies that did not fall into either of the above categories but had a primary aim of exploring how ≥2 social characteristics intersect to influence nutrition outcomes.

These categories were required in the theoretical framing of the analysis, aim, title, or abstract and should not have been used solely for interpreting results. The detailed inclusion and exclusion criteria are available in Supplemental Methods 2.

Although we used “inequity” as a search term in our review, the majority of quantitative studies retrieved focused on inequalities rather than the systemic reasons behind these differences (inequities). Consequently, the term “inequity” would not be applicable to most of the studies included in our review. Therefore, to maintain consistency and accurately reflect the reviewed literature, we focus on “equality” throughout our review article.

#### Data screening and extraction

We imported citation results into Eppi Reviewer Web (Version: 4.12.10) and excluded duplicates. EF screened titles and abstracts and full texts. A second reviewer (MZ) independently screened 20% of titles and abstracts and all full texts. Conflicts were resolved by a third reviewer (SK or HHF). We extracted data using a pretested form (50% checked by a second reviewer [CO]). The extracted data were article characteristics (author and publication year), characteristics of the research (category of engagement with intersectionality, data source, study design, and sample sizes), characteristics of the study population (country, age, gender, and any other focal characteristics for inclusion in the study), nutrition outcomes, social characteristics included in intersectional investigations, explanatory mechanisms explored (if applicable), statistical methods including covariate adjustment, and key findings.

Based on definitions provided by Bauer and Scheim (2019) [16], we classified approaches to intersectionality into 2 categories: descriptive and analytic. With descriptive intersectionality, the intersections are treated as the primary predictors for nutrition outcomes. With analytic intersectionality, mechanism(s) that may contribute to or explain intersectional inequalities in nutrition outcomes are also investigated. As mechanisms may contribute toward inequalities in nutrition by either being unequally distributed across intersecting social characteristics (differential exposure) or by having heterogeneous effects on nutrition outcomes across intersectional groups (differential effects) [25], we further classified analytic approaches as being focused on differential exposure, differential effects, or both. A glossary of key terms used in our review is shown in Table 1.

**TABLE 1:** Glossary of key terms

Term	Description
Intersectionality	A framework for understanding how multiple systems of inequality based on social characteristics such as race, gender, religion, disability, and class intersect to shape unique experiences of discrimination and privilege.
Intersectional groups/intersections	A group of individuals characterized by at least 2 of their social characteristics.
Mechanism	An intermediate variable (that is neither a social characteristic of the intersectional groups nor a nutrition outcome) that represents a social process or aspect of society that may contribute to or explain intersectional inequalities in nutrition outcomes.
Descriptive intersectionality	An investigation in which intersections are treated as the primary predictors for nutrition outcomes, i.e., mechanisms are not investigated.
Analytic intersectionality	An investigation that explores how a mechanism(s) may contribute to or explain intersectional inequalities in nutrition outcomes, either through differential effects, differential exposure, or both.
Differential effects hypothesis	An investigation that explores heterogeneity in the effect of a mechanism(s) on nutrition outcomes across intersectional groups.
Differential exposure hypothesis	An investigation that explores whether differences in the level of exposure (or distribution) of a mechanism(s) across intersectional groups cause or partially explain intersectional inequalities in nutrition outcomes.
Additive, nonadditive/departs from additivity	The additive assumption implies the effects of social characteristics can be understood as a sum of their parts. For example, the effects of being a female from a disadvantaged class = female + disadvantaged class. However, intersectionality posits that social characteristics are mutually constituted, not independent, and combine in a way that is nonadditive/departs from additivity. For example, the effects of being a female from a disadvantaged class ≠ female + disadvantaged class.
Additive scale interaction	The default interaction scale for linear regression, which assesses whether the combined effect of the 2 exposures is different from the sum of the independent effects of the 2 exposures. In other words, additive scale interactions assess departure from additivity (e.g., female + disadvantaged class ≠ female from disadvantaged class) and, therefore, have relevant interpretations to intersectionality.
Multiplicative scale interaction	The default interaction scale for nonlinear (e.g., logistic, log-binomial, Poisson) regression, which assesses whether the combined effect of the 2 exposures is different from the product of the independent effects of the 2 exposures. In other words, multiplicative scale interactions assess departure from multiplicativity (e.g., female × disadvantaged class ≠ female from disadvantaged class).

### Analysis

Characteristics of descriptive and analytic studies are shown in tables and figures. We narratively describe key trends for findings from descriptive studies. For analytic studies, study findings are shown in tables, and in-text examples are used to illustrate key points.

## Results

**FIGURE 1:**
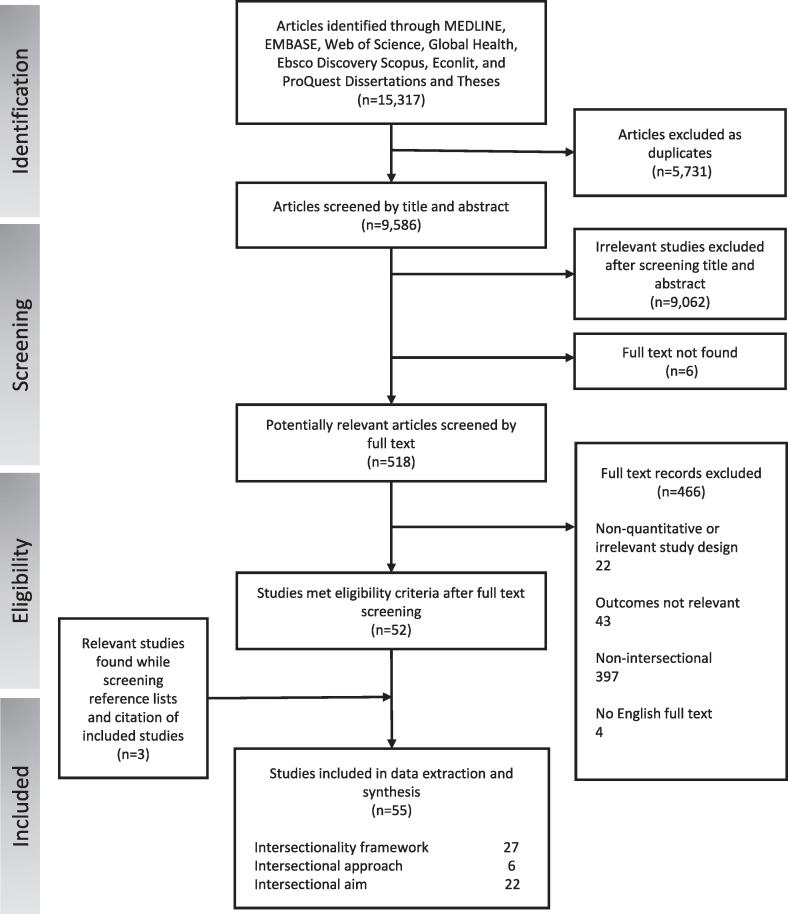
Study flow diagram for the review process.

The PRISMA flowchart shows our study selection process (Figure 1). Of the 15,317 results retrieved from our search strategy, 55 articles were included in our review [[36], [37], [38], [39], [40], [41], [42], [43], [44], [45], [46], [47], [48], [49], [50], [51], [52], [53], [54], [55], [56], [57], [58], [59], [60], [61], [62], [63], [64], [65], [66], [67], [68], [69], [70], [71], [72], [73], [74], [75], [76], [77], [78], [79], [80], [81], [82], [83], [84], [85], [86], [87], [88], [89], [90]]. Supplemental Table 1 provides an overview of study characteristics. Trends show an increase in studies since 2015, with just 15% of studies published before then.

### Where has the research taken place?

Studies featured populations in 14 countries: 41 (75%) in high-income, 5 (9%) in upper-middle income, 9 (16%) in lower-middle income, and 1 (2%) in a low-income country (Mozambique). At the national level, only populations in the United States (*n* = 35) and India (*n* = 7) were represented by >2 studies. One study featured populations from 2 countries (Mozambique and China) and so studied different regions and income quartiles, and 1 featured populations from 2 countries (Canada and the United States) in the same region and income quartile (Supplemental Table 1).

### What are the characteristics of the study populations?

Most studies analyzed populations at the national level (65%). One study featured men only [87], and 5 only studied women [44,47,74,75,90]. Around half the studies restricted their sample to specific racial or ethnic groups, and 7 noted excluding particular racial and/or ethnic groups due to insufficient sample sizes [38,41,47,48,69,76,81]. Other specific study population characteristics included adults with a disability [55], adults from sexual minority groups [47], employed adults only [61], and low-wage employed adults only [56].

Nutrition outcomes were primarily assessed among adults, with 7 studies focusing on older adults (mostly ≥50 y) [42,51,53,68,74,78,89]. Six studies focused on nutrition outcomes among children and adolescents (aged 5–17 y) [45,46,54,59,60,88]; 7 focused on young children (aged <5 y) [37,49,66,[70], [71], [72],^86^]; and 1 study focused on nutrition outcomes among individuals spanning both these age categories (aged 2–15 y) [60]. Three studies explored nutrition outcomes from adolescence through to adulthood [64,77,85], and 1 focused on both adults (mothers) and young children [67]. The remaining studies explored nutrition outcomes at the household level [39,62,75,79,84] (Supplemental Table 1).

### Which nutrition outcomes have been studied?

**FIGURE 2:**
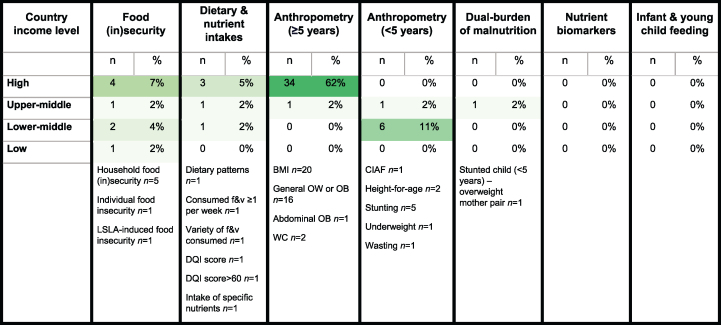
Heat map of nutrition outcomes assessed in the included articles by country income level. Dietary and nutrient intakes were evaluated among adults only. BMI, body mass index; CIAF, composite indicator of anthropometric failure; DQI, diet quality index; f&v, fruit and vegetables; LSLA, large-scale land acquisition; OB, obesity; OW, overweight; WC, waist circumference.

The frequency of nutrition outcomes assessed is shown in Figure 2. Anthropometric measurements among populations aged ≥5 y were most common and only investigated in high and upper-middle income settings. Anthropometry among children aged <5 y was explored in 7 studies in upper-middle and lower-middle income settings [37,49,66,[70], [71], [72],^86^]. Indicators of food security were investigated in 7 studies across settings of all income levels [39,43,62,73,75,79,84]. Dietary and nutrient intakes were investigated among adults only and across 5 studies [36,44,53,74,82] that covered countries of all income levels besides low income. One study investigated a dual burden of malnutrition (stunted child and overweight mother pair in Mexico) [67]. To our knowledge, no studies investigated nutrient biomarkers or infant and young child feeding practices.

### Which social characteristics and intersectional relationships have been studied?

Thirteen broad social characteristics were included within intersectional enquiries. Race or ethnicity was most common (*n* = 36; 65%). Around 50% of studies that included race or ethnicity referred to “race/ethnicity” to either describe racial-ethnic categories, categories of race only, or race and ethnicity were used interchangeably; hence, hereafter, we refer to race and/or ethnicity. Race and/or ethnicity were included in all studies based in the United States but just 2 outside the United States [78,89]. The next most common was gender (*n* = 33; 60%), and the least common was indigeneity (studied only in Guatemala [86]) and religion (studied only in India [70]). Other social characteristics studied exclusively in certain countries included sexual orientation (United States) [45,[47], [48], [49], [50],^59^,^75^] and caste group (India) [37,49,62,66,[70], [71], [72]] (Supplemental Table 1).

**FIGURE 3:**
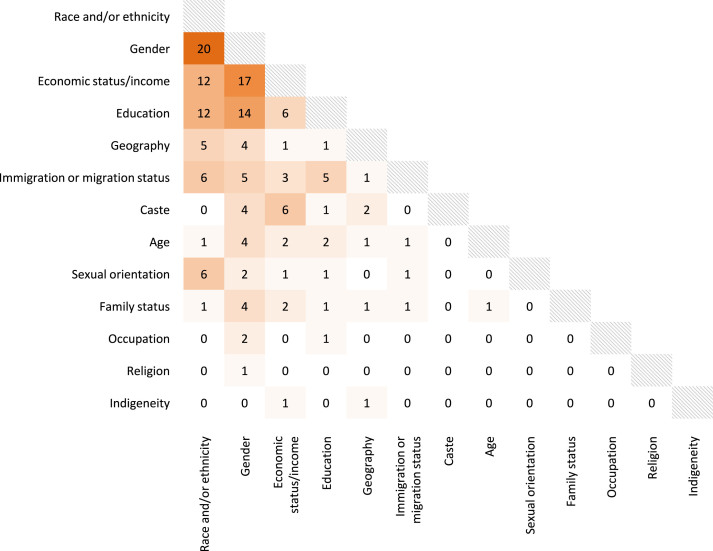
Intersectional relationships between social characteristics investigated in the included articles. Family status includes civil/marital status and living arrangements, such as living alone or with a partner. The data underlying this figure are shown in Supplemental Table 2.

Figure 3 shows the intersectional relationships investigated between social characteristics in the included studies (data in Supplemental Table 2). Intersections of race and/or ethnicity and gender were most common (*n* = 20), followed by gender and economic status/income (*n* = 17). Most analyses intersected 2 characteristics at a time; 3 studies intersected ≥5 simultaneously [40,58,59].

### Which study designs have been implemented?

Studies mostly used secondary data, which came from >30 sources (not accounting for the different survey rounds) (data sources shown in Supplemental Table 3). Although no data were mentioned as collected specifically for intersectional analyses, the author, using data from The Chicago Health and Life Experience of Women, noted convenience sampling of lesbian women in the first wave and included a supplemental sample of bisexual women, younger women, and Black and Latina women in the third wave [47]. Additionally, authors [38,[57], [58], [59],^64^,^73^,[75], [76], [77],^87^] using 6 different data sources, reported oversampling of minority groups. Of particular interest, 2 studies [76,77] using the National Longitudinal Study of Adolescent Health in the United States, reported the oversampling of an intersectional group: Black adolescents with at least 1 college-educated parent.

All included studies used solely quantitative methods (i.e., none used mixed research methods). Most (*n =* 44; 80%) featured cross-sectional study designs, and 11 featured longitudinal observational study designs. Of the longitudinal studies, 5 explored whether intersectional inequalities in nutrition outcomes change across the life course [38,63,64,68,85]; 2 contrasted intersectional group-specific changes in nutrition outcomes between time points [41,42]; and 3 focused on intersectional inequalities in nutrition outcomes at a single time point based on social characteristics measured before then [53,76,88]. One study employed a quasi-experimental design, and we did not identify any other experimental or intervention-focused research. More than 50% of studies only provided estimates adjusted for other social characteristics beyond those of the focal intersectional groups. We classified most studies (*n =* 46; 84%) as descriptive intersectionality and 9 (16%) as analytic (Supplemental Table 1).

### What methods have been used to describe intersectionality in nutrition outcomes, and what are the key findings?

Statistical methods used for descriptive approaches to intersectionality and corresponding citations are shown in Supplemental Table 4. Regression with interaction terms between social characteristics was most common (*n* = 26): 14 used linear regression and tested for interaction on the additive scale, and 15 used nonlinear regression. Of those using nonlinear regression, 11 tested for interaction on the multiplicative scale only, 1 tested for interactions on the additive scale only, 1 tested for interactions on both the additive and multiplicative scale, and 2 did not report the interaction but instead gave the predictive probabilities of the nutrition outcome for each intersectional group.

Another common approach included regressing the nutrition outcome on a variable where the levels represent different intersectional groups (e.g., variable coded as: 0 = White male; 1 = White female; 2 = Black male; and 3 = Black female) (*n* = 11). Three studies used an approach conceptualized by Sen et al. (2019) [27], which instead regresses the outcome on multiple intersectional variables (e.g., var1: 1 = White male; 0 = not White male; var2: 1 = White female; 0 = not White female, etc.), which then allows chi-squared tests to make comparison across the entire social spectrum, as opposed to just the base category. Four studies used stratified regression only.

Five studies applied one of the above methods to growth curve models to assess how intersectional inequalities in nutrition outcomes diverge across the life course, and 4 used a multilevel analysis of individual heterogeneity and discriminatory accuracy, a predictive modeling approach conceptualized for the study of intersectionality that aims to understand how much of the intersectional group variation in the outcome is captured by the additive main effects of the social categories (i.e., nonintersectional) or their interaction effects (i.e., “intersectionality” effects).

For studies using nonregression-based methods, 2 employed descriptive methods only, and 2 used a decomposition analysis method (Corrected General Entropy Class Index) to compare whether the amount of between-social group inequality as a portion of the total inequality in the nutrition outcome is greater when intersectional groups are considered (e.g., caste by wealth groups) compared to a single social category (e.g., only wealth)**.**

Evidence from the United States demonstrated differences in nutrition outcomes at the intersection of race and/or ethnicity and gender. For instance, one study showed how the combined influence of being Black and a woman was associated with a higher average BMI than the sum of the independent effects of the 2 identities [59]. Two studies showed how White women were less likely, whereas Black women were more likely, to be overweight than men of the same race [65,83]. Further, 2 studies found that the association between skin tone and BMI also varied by gender, with darker skin tones associated with elevated BMI and odds of obesity among women but not men [63,76]. Evidence also showed how race and/or ethnicity intersect with economic status, with several studies demonstrating that higher economic status was associated with better nutrition among racial and/or ethnic majorities (e.g., White populations) but not, or to a lesser extent, among minorities (e.g., Black or Hispanic ethnic groups) [51,55,64].

In India, evidence demonstrated that higher economic status was protective against child undernutrition, regardless of caste or gender [49,66,71]. However, the combination of caste and economic status had different effects on gendered-nutrition inequalities. For poorer and more marginalized castes, there was either no difference by gender or boys were more likely to be undernourished. As social advantage increased in either dimension, the burden of child undernutrition shifted toward girls [70,72]. Further, evidence showed how among Hindus, child undernutrition is higher among girls than boys, whereas among non-Hindus, the association with gender operates in the opposite direction [70].

The remaining 12 countries included had insufficient evidence for narrative syntheses (≤2 studies per country). Several studies with similarities at that regional level were synthesized and shown in Supplemental Results 1.

### What methods have been used to study analytic intersectionality, and what are the key findings?

Details of the studies classified as analytic are shown in Table 2 [41,42,43,56,63,69,76,77,90]. Fifty-six percent featured longitudinal study designs, and 44% incorporated control variables into analyses. One longitudinal study employed a quasi-experimental design to identify how the effects of adolescents’ peer social contexts on obesity differ across intersectional groups. To specifically attribute these effects to the social context, the authors incorporated controls to approximate random assignment of adolescents to social contexts within schools [77].

**TABLE 2:** Characteristics and findings of studies classified as analytic approaches to intersectional inequalities in nutrition

Author [reference number]	Social characteristics	Mechanism(s)	Nutrition outcome	Method used	Confounding considerations^1^	Key findings
Differential exposure						
Hargrove et al. [63]	Race (interviewer-ascribed skin tone); gender	Income; education; marital status; chronic strains; experiences of racial or color discrimination-; various health behaviours^2^,^2, 2^	BMI at age 32 years	Regression with Informal Mediation Assessment	—	• The association between increasingly darker skin tone and greater average BMI varies by gender; evidence of association among women but not men. • No evidence that the mechanisms explained these trends.
Hargrove et al. [63]	Race (interviewer-ascribed skin tone); gender	Income; education; marital status; chronic strains; experiences of racial or color discrimination; various health behaviors^2^	BMI trajectories between ages 32 and 55 years	Regression with Informal Mediation Assessment	—	• BMI increased less among medium than lighter and darker skin-toned men, whereas BMI gaps between lighter and darker skin-toned women remained consistent. • Higher education among medium skin-toned men explained ∼14% of the divergence in BMI among men. • No evidence that any of the mechanisms explained the consistent increases in BMI among women of all skin tone categories.
Atuoye et al. [43]	Gender; migration status	Perceived climate change stressor	Large-scale land acquisition-induced (LSLA) food insecurity.	Regression with Informal Mediation Assessment	Yes (socioeconomic, bio-socio-cultural and locational factors)	• Compared with nonmigrant females, nonmigrant males and migrant males and females were more likely to report that LSLA are resulting in food insecurity. When accounting for perceived climate change stressors: • The impact of LSLA on food insecurity was reduced among nonmigrants but exacerbated among migrants. • Differences only remained between male and female nonmigrants.
Perreira et al. [76]	Race (interviewer-ascribed skin tone); gender	Perceived stress, discrimination, income and economic hardship	Obesity; BMI	Regression with Sobel tests for indirect effects	Yes (foreign-born, married or cohabiting, urban location, health insurance coverage, and United States region)	• The associations between increasingly darker skin tone and greater odds of obesity and average BMI vary by gender; evidence of association among women but not men. • The association among women is partially mediated by income and economic hardship but not perceived stress or discrimination.
Differential effects						
McClain et al. [69]	Racial-ethnic group; gender	Subjective social status (SSS)	BMI; WC	Regression with interaction terms between social characteristics and mechanism	Yes (childhood household income, maternal education, adulthood household income, educational attainment)	• Evidence of 3-way interaction between SSS, racial-ethnic group, and gender on BMI and WC. • The association between increased SSS and lower BMI and WC is greater among non-Hispanic White women than White men but does not vary by gender for other racial-ethnic groups. • The association between increasing SSS and BMI and WC varies by racial-ethnic group among women but not men; stronger positive association among non-Hispanic Black than non-Hispanic White females.
Polos et al. [77]	Race; gender; adolescent household income level	Adolescent peer economic disadvantage	Obesity	Regression with interaction terms between social characteristics and mechanism^3^	Yes (several controls to reduce endogeneity bias and isolate quasi-experimental variation in the prevalence of the mechanism)	Relationships between adolescent peer economic disadvantage and obesity for each intersectional group^3^: • Black men of lower-income households: Strong negative relationship which strengthens over time; • Black women of all household income levels: Strong positive relationship that emerges after high school; • Non-Black women of all household income levels: Modest positive relationship; • Non-Black men of all household income levels: No relationship.
Assari et al. [41]	Race; gender	Education and income	Sustained high BMI over 6 years	Multigroup SEM	Yes (effects for education adjust for income; effects for income adjust for education)	• Protective effect of higher education against sustained high BMI among White men and women, but not Black men and women. • Protective effect of higher income against sustained high BMI among Black and White women, but not Black and White men.
Assari et al. [42]	Race; gender	Education > income	Changes in BMI over 6 years	Moderated mediated using multigroup SEM (latent growth curve modeling)	—	• Protective effect of higher education against changes in BMI for White men, but not White women or Black men and women. • No evidence that income mediates the protective effect of higher education against changes in BMI among White men.
Bell et al. [90]	Race; education	Weight underestimation	BMI	Moderated mediation using the counterfactual framework	—	• Black women had a higher BMI than White women, and inequalities were more substantial in college than non-college graduates. • Indirect association between race and BMI through weight underestimation found among non-college but not college graduates. • Race differences in BMI still remained after accounting for weight underestimation among college and non-college graduates.
Differential exposure and differential effects						
Durfee et al. [56]	Race; gender; geography	Several personal and policy-level determinants of BMI^4^	BMI	Oaxaca-blinder decomposition	—	• Average BMI for Black women differs between Raleigh and Minneapolis. • Differences in age and education (different exposure) explained 97% of the geographic difference in BMI. • Virtually none of the gap is attributed to differential treatment (differential effects) of Black women across the 2 cities.

Abbreviations: BMI, body mass index; LSLA, large-scale land acquisition-induced; SEM, structural equation modeling; SNAP, Supplemental Nutrition Assistance Program; SSS, subjective social status; WC, waist circumference.

1 None if only controlled for age.

2 “Chronic strains” is an indicator made up of 4 domains: health of close others, work, finances, and relationships; ‘health behaviors’ includes 4 behaviors: heavy alcohol use, smoking status, fast food consumption, and physical activity.

3 Interaction term not reported – results are postestimation marginal effects.

4 Personal and policy-level determinants of BMI included: ethnicity, age, probability of education above high school, and access to resources (including SNAP recipiency, food insecurity, access to credit, physical activity polynomial, hourly wage). Four studies used cross-sectional study designs (McClain et al. [69]; Durfee et al. [56]; Bell et al. [90]; Atuoye et al. [43]), and the remaining studies used longitudinal study designs.

*Differential exposure hypotheses* (*n* = 3): Two studies [43,63] used regression models with informal mediation assessment, which involves observing changes to intersectional inequalities in nutrition outcomes once variables representing the mechanisms of interest are additionally included in the regression model. For example, cross-sectional evidence from Tanzania showed that the probability of reporting food insecurity induced by large-scale land acquisitions was greater among nonmigrant males than nonmigrant females and migrant males and females, but these differences were not observed once climate change stressors were accounted for [43]. Another study from the United States [76] expanded on this methodology by statistically testing for mediation (using Sobel tests for indirect effects) and found an association between darker skin tone and greater odds of obesity and average BMI among women, but not men, and that the association among women was partially mediated by racial differences in income and economic hardship but not perceived stress or discrimination [76].

*Differential effects hypotheses* (*n* = 5): Two studies used regression models with interaction terms between multiple social characteristics and mechanisms of interest [69,77]. For example, Polos et al. (2021) [77] found that the effects of adolescent peer economic disadvantage (the mechanism) on adulthood obesity varied across intersections of race, gender, and parental income in adolescence in the United States. Another 2 studies used multigroup structural equation modeling, where models were stratified by intersectional groups to investigate differential effects of the mechanisms on the nutrition outcomes [41,42]. To illustrate, Assari et al. (2019) [42] showed how the protective effects of higher education (the mechanism) against changes in BMI over 6 y among older adults in the United States differed across race by gender intersections, with an association found only among White men. Assari et al. [42] then extended this approach to moderated mediation by exploring whether the association among White males was mediated by income but found no evidence to support this hypothesis. Another study based in the United States also applied moderated mediation but used a counterfactual framework to show how the mediating role of weight underestimation (the mechanism) in racial inequalities in BMI differed by education level, with an indirect association found among noncollege graduates but not college graduates [90].

*Differential exposure and effects hypotheses* (*n* = 1): The sole method identified was an Blinder–Oaxaca decomposition, used to investigate why the combination of being Black and a woman was associated with a higher average BMI in one United States city compared with another [56]. BMI differentials were decomposed to assess the portion of the inequality that was due to differences in the levels of several mechanisms (differential exposure) or differences in the effects of the mechanisms on BMI (differential effects). Differences in average age and education level explained 97% of the inequality, and therefore, it was concluded that virtually none of the BMI inequality is attributable to the differential treatment of Black women between the 2 cities.

## Discussion

Our systematic scoping review aimed to assess where and how quantitative nutrition research is engaging with intersectionality and identify gaps in the evidence and implementation of its approach. Of the 55 articles included, most were published since 2015. This reflects increasing attention toward the complexity of inequalities in nutrition following the SDG’s commitments to address malnutrition and leave no one behind [8].

We found that race or ethnicity were the most common social characteristics and were most often intersected with gender, consistent with other reviews [23,91]. The high prevalence of race within intersectional investigations reflects the origins of intersectionality in critical race theory [92]. However, we found that the literature often conflated concepts of race and ethnicity, with many studies using the terminology “race/ethnicity.” To prevent overlooking the different ways in which race and ethnicity intersect, the conflation of these concepts must be avoided in future research. Future research must also consider a greater breadth of historically oppressed social characteristics, such as indigeneity, religion, displaced persons, and persons with disability. Additionally, although stigma associated with some sexual orientations is known to increase vulnerability to inequitable health outcomes globally [93], sexual orientation was only investigated among studies in the United States. Although this gap needs to be addressed, creating the required data infrastructure may not be equally feasible across contexts due to severe stigmatization and sometimes criminalization of nonheterosexual behaviors and identities.

In both the United States and India, studies revealed the importance of an intersectional lens for a more accurate understanding of inequalities in nutrition outcomes. In the United States, studies demonstrated interdependent relationships between different “horizontal groups,” which draws attention to discrimination based on oppressed characteristics [94]. For example, racial inequalities in nutrition outcomes were shown to depend on gender and vice versa. Additionally, nutritional experiences of “vertical groups” (which are more amenable to change, such as education and income [94]) were shown to differentiate between horizontal groups. For example, in the United States, the protective effects of higher economic status against a higher BMI were shown to be reduced among racial and ethnic minorities than majorities. In India, the evidence demonstrated how gender inequalities in child stunting vary based on religion and across intersections of caste and economic status, underscoring the nuances that intersectionality analyses reveal, as average gender differences in child stunting are rarely observed in India [95].

Despite the value of intersectionality inquiry for nutrition in low- and middle-income countries (LMICs) for expanding the commitment to “leave no one behind” [96,97], <35% of the evidence came from populations in LMICs. Considering the high rates and inequitable distribution of food insecurity and undernutrition in many LMICs, this needs to be corrected with future research. For example, Indonesia, Ethiopia, Nigeria, and Kenya, none of which were represented in our review, have been identified as having greater inequalities in child wasting across communities than any other countries, with rates varying up to 9-fold [7]. If estimates were to consider how different axes of marginalization intersect within these countries, much starker trends may be revealed. Further, several important indicators were omitted, including indicators of undernutrition among adults, infant and young child feeding, and micronutrient status. To make progress in reducing intersecting inequalities in nutrition outcomes, the entire spectrum of pressing public health nutrition challenges needs to be considered.

The absence of evidence using mixed methods is a limitation of the current nutrition literature. As quantitative approaches to intersectionality gain traction, qualitative methods should not be neglected. Rigorous mixed-methods research can help answer complex intersectionality-based research questions in nutrition and identify intervenable mechanisms that contribute toward disparate outcomes [21].

Another area that requires further attention is the inconsistency in analysts’ inclusion of covariates. Around half of the reviewed studies only provided estimates adjusted for confounding (and potentially mediating) social characteristics, with the presumed aim of isolating the direct effects of discrimination against people with a particular set of intersectional characteristics from the effects of other forms of disadvantage [98]. On the other hand, around half of studies provide unadjusted estimates, and these typically aim to represent the total (sometimes termed “structural”) effects of discrimination. Unlike direct effects, these structural estimates include the effects of intersecting characteristics on nutrition outcomes that arise through mediating effects on other characteristics, such as the combined effects of racism and sexism on nutrition that occur through income inequality. A challenge for the analyst is thus to appropriately distinguish confounders from mediators—a challenge that was not well addressed in most studies in our review. The debate about whether and what covariates to include in inequality measures is not new [[99], [100], [101]]. However, when done well, comparisons of crude and adjusted estimates can be useful for identifying the most nutritionally disadvantaged populations and quantifying the harms of particular forms of discrimination. Future research should ensure that intended estimands (direct or structural effects) and risk of bias are clearly described so that estimates can be correctly interpreted [102]. Furthermore, for evidence to inform intervention design, the ability to infer causality between the mechanism and the nutrition outcome is critical [25]. However, just one included study classified as analytic mentioned taking specific measures to address the risk of confounding—an important gap in the nutrition literature.

A further related limitation is that most (80%) studies relied on cross-sectional datasets. These datasets are suitable for descriptions of inequality within horizontal social characteristics but are less suited to studies of how vertical social characteristics change over time or studies aiming to distinguish interactions from processes (e.g., is racism interacting with poverty or causing it?). More evidence with longitudinal data is needed to understand how the accumulation of vertical characteristics varies across intersections of horizontal characteristics and the implications of this for nutritional health, particularly in settings where malnutrition rates are high but good-quality data are scarce [103].

Our review showed how a wide range of secondary data sources are being used to explore intersecting inequalities in nutrition outcomes. Yet, most of these data are not collected for intersectionality analyses. Commonly used proportion-to-population sampling means low representation of individuals with one or multiple minority characteristics. In several studies, this resulted in certain minority groups being excluded or having particularly small sample sizes. These challenges advocate for intersectional approaches to data systems that represent the needs of the most nutritionally vulnerable groups, which current datasets rarely facilitate [104].

In terms of methods used, regression analysis with interaction terms between social characteristics was most common. When interactions were tested from linear regression models, methods aligned with intersectionality by allowing social positions to depart from additivity [22,29]. However, among studies testing interactions from nonlinear models (e.g., logistic or Poisson), only 2 of 13 studies took extra steps to assess interaction on the additive scale—the more appropriate method for quantitatively engaging with intersectionality [22,28]. Because additive interaction is relevant for assessing the public health impact of the intersecting social characteristics when the outcome is discrete, i.e., excess absolute cases due to the presence of the interaction [31], we recommend that future research reports and correctly interpret interactions from nonlinear models on both the multiplicative and additive scale [31].^.^

Most studies identified as analytic focused either on differential effects hypotheses or differential exposure hypotheses. Although both these investigations can help inform the design of interventions, when investigating one or the other, it is essential to consider that both may be occurring simultaneously [105]. For example, multiply marginalized groups may have restricted access to a nutrition service (differential exposure), but when these groups access that service, the relative benefits to their nutrition status may differ from the less marginalized groups (differential effects). This is especially important for modeling potential benefits of an intervention—if the mechanisms by which an intervention works also have heterogeneous effects on the outcome that are not accounted for, then the estimated disparity reduction under a hypothetical intervention that eliminates disparities in the mechanisms will be inaccurate. The study using an Blinder–Oaxaca decomposition was the only one to use a methodology that accounts for this complexity, although other methods are available, such as those that allow for exposure–mediator interactions [106].

Finally, few analytic studies investigated mechanisms tightly interlinked with factors that are intervenable within the public health nutrition policy space, such as aspects related to the food environment, nutrition education, and the role of existing nutrition policies and programs. To produce equitable health outcomes, it is essential to understand the varied impacts of nutrition policies and programs on different groups, at minimum, to ensure that initiatives do not adversely affect health equity [3]. To aid this, an intersectionality-based policy analysis framework was developed by Hankivsky et al. [107]. However, there is a lack of resources on best practices for integrating intersectionality into intervention research, as well as a lack of health intervention research doing so [108]. In Box 1, we highlight recommendations and priorities for future quantitative nutrition research engaging with intersectionality.

Box 1 **Recommendations for quantitative nutrition research engaging with intersectionality**

1. **Improve data systems in nutrition across all countries:**
  a. To enable adequate sample sizes for intersectional analyses by identifying and oversampling minority intersectional groups.
  b. To understand temporal trends in inequalities in nutrition outcomes and bridge gaps among understudied intersectional groups and contexts by collecting and analyzing high-quality and longitudinal data.
2. Advance quantitative methods:
  a. To include rigorous mixed-methods studies that can provide a more comprehensive understanding of how and why intersecting social characteristics shape nutrition outcomes.
  b. To ensure alignment of hypotheses and methods, particularly when: *1*) using interaction terms in nonlinear regression, and *2*) using covariate adjustments.
  c. To disentangle the ways by which interventions and policies can widen or narrow nutrition inequalities through inequalities in both inclusion and pathways to impact.
3. **Use theory** to conduct research that focuses on identifying mechanisms that can be intervened on to reduce intersectional inequalities in nutrition outcomes.
4. **Widen the scope** to consider a breadth of nutrition outcomes, historically oppressed social characteristics, and contexts.
5. **Evaluate existing nutrition policies** and interventions to understand the inclusivity and equity of coverage and nutrition outcomes.

Alt-text: Box 1

### Strengths and limitations

Strengths of our review include using 8 databases to capture the breadth of both peer-reviewed and gray literature and using PRISMA-ScR guidelines to ensure a replicable and rigorous review process. Additionally, in contrast to other reviews [20,28,91], we included quantitative nutrition research that incorporated concepts of intersectionality but without explicitly referencing the framework. This is important because although attention to intersectionality in public health has only recently gained traction, research may have already embodied its principles without mentioning them specifically. However, a limitation is that relevant studies may have been excluded if the study aim was not explicit or unclear. Other limitations include our reliance on articles in the English language, potentially excluding relevant articles in other languages; having only 1 reviewer screen all titles and abstracts against our inclusion criteria, with the second reviewer reviewing <50%; and our lack of quantitative syntheses due to the breadth of the review and the resulting diversity of included studies. Finally, because a core focus of intersectionality is how an individual’s multiple characteristics intersect within macro-level systems of inequality, articles using community-level characteristics (e.g., measures of community-level deprivation) to indicate intersectional groups were excluded. However, we included studies where community-level characteristics were used to indicate potential drivers or mechanisms of intersectional inequalities in nutrition outcomes. We note that not all research engaging with intersectionality may follow this criterion but must be cautious to ensure that the agency of the individuals experiencing interlocking forces of power and oppression is not undermined in this inquiry.

## Conclusion

This systemic scoping review describes the emerging evidence on quantitative nutrition research engaging with intersectionality. Going forward, improved data systems that facilitate intersectional analyses will be integral for advancing this research agenda and ensuring the experiences and needs of the most oppressed and marginalized groups are understood. Furthermore, there is a need for innovations in research methodologies with an emphasis on mixed-method and intervention-oriented research that provides comprehensive insights into the structural and societal drivers of intersectional inequalities in nutrition outcomes and strategies to address them.

## Author contributions

The authors’ responsibilities were as follows – EF: conceptualized the systematic scoping review with inputs from HH-F, SK, EA, BS, EF: conceived and ran the search strategy; EF, MZ: screened references and selected eligible studies based on the eligibility criteria; EF: extracted data from included studies; CO: checked data for accuracy; EF: wrote the manuscript with substantial inputs from HH-F, SK, EA, BS, EF: had primary responsibility for the final content; and all authors: read and approved the final manuscript.

## Conflict of interest

The authors report no conflicts of interest.

## Funding

Funding of EF, CO, SK, BS, and MK was provided by Innovative Methods and Metrics for Agriculture and Nutrition Actions (IMMANA). IMMANA is co-funded with FCDO from the UK Government (grant number 300654) and the Bill & Melinda Gates Foundation (INV-002962/OPP1211308). Funding of author HH-F was provided by a Sir Henry Wellcome grant (210894/Z/18/Z). These funders had no role in the design, implementation, analysis and interpretation of the data.

## Data availability

All data are included in this article or are available in the supplementary file.
