# Supplementary material for: The Extent, Range, and Nature of Quantitative Nutrition Research Engaging with Intersectional Inequalities: A Systematic Scoping Review
Source: Adv Nutr. 2024 May 4;15(6):100237. doi: 10.1016/j.advnut.2024.100237 (PMC11180316; doi:10.1016/j.advnut.2024.100237)
Supplement: Multimedia component 1 [file mmc1.docx]

**Supplemental Methods 1**. Example search strategy for Ovid MEDLINE.

| 1 | (Underweight or overweight or adipos* or "fat mass" or "lean mass" or "body composition" or BMI or "mid-upper arm circumference" or "mid upper arm circumference" or MUAC or "body mass index" or "body-mass index" or obes* or weight-for-height or weight-for-length or WHZ or WLZ or stunt* or HAZ or height-for-age or length-for-age or wasting or wasted or anaemi* or anemi* or h?emoglob* or undernutri* or overnutri* or undernour* or overnour* or malnutri* or malnour* or thinness or "nutritional status" or nutrition or "food secur*" or FIES or "food share" or "food consumption score*" or HFIAS or (food adj5 (expenditure*)) or (food adj3 (share*)) or MAHFP or "household dietary diversity score*" or HDDS or diet* or micronutrient* or vitamin* or anthropom* or IYCF or breastfeeding or "breast feeding" or breastfed or "infant feeding").ti,ab,kw. |
| --- | --- |
| 2 | thinness/ or exp overweight/ or exp anemia/ or exp nutrition disorders/ or exp obesity/ or body mass index/ or nutritional status/ or skinfold thickness/ or anthropometry/ or exp micronutrients/ |
| 3 | or/1-2 |
| 4 | intersectionality.mp. |
| 5 | ((intersect* or inter-secti* or Interact* or "joint effect*" or joint-effect* or "joint dispsarit*" or overlap* or inter-connect* or "inter connect*" or interconnect*) not intersectoral).ti,ab,kw. |
| 6 | (inequalit* or equalit* or equit* or inequit* or marginal* or (social adj2 (categor* or group*)) or minorit* or disparit* or "Social determinant*" or "socioeconomic dimension*" or ((Sociological or social) adj2 factor*) or disadvantage* or "vulnerable group*" or "sociodemographic characteristic*").ti,ab,kw. |
| 7 | socioeconomic factors/ or exp social classes/ or health status disparities/ or health equity/ or minority groups/ or social marginalization/ or "social determinants of health"/ or vulnerable populations/ |
| 8 | or/6-7 |
| 9 | 5 and 8 |
| 10 | 4 or 9 |
| 11 | 3 and 10 |

**Supplemental Methods 2.** Systematic scoping review inclusion/exclusion criteria.

| Inclusion Criteria | Exclusion Criteria |
| --- | --- |
| Population | |
| All human populations. | Animal studies/biological studies on human tissue. |
| Methodology | |
| Any quantitative research methodology, including mixed-method and program evaluation studies. | Studies using exclusively qualitative methods, or KAP (knowledge, attitude and practice studies). |
| Nutrition outcome | |
| Anthropometric measurements, micronutrient status, food or nutrient consumption/intake, infant and young child feeding practices (e.g., breastfeeding duration or initiation, exclusive breastfeeding up to 6 months, or minimum acceptable diet), and indicators of food security (including aspects of food security such as food availability or access) pertaining to the individual or household. | Aggregate indicators where a nutrition outcome is included amongst other non-nutrition outcomes, perceptions of body image and eating behaviours. |
| Intersecting social characteristics | |
| Nutrition outcomes or inequalities are assessed based on at least two intersecting social characteristics pertaining to the individual or household (e.g., social group affiliation, geography/place of residence, economic status, gender, race, and ethnicity). | Community-level characteristics that are indirect to the individual or households (e.g., measures of community-level deprivation, fast food outlet density, or other characteristics of the environment in which one lives). |
| Engagement with intersectionality | |
| Fell into one of the following three categories:   1. Framework: Studies that explicitly referenced the intersectionality framework 2. Approach: Studies that stated taking an ‘intersectional approach’, or used other similar terminology, such as ‘intersectional analysis’ or ‘intersectional inequalities’, but did not refer directly to the intersectionality framework. 3. Aim: Studies that did not fall into either of the above categories but had a primary aim of exploring how two or more social characteristics intersect to influence nutrition outcome   These categories were required in the theoretical framing of the analysis, aim, title or abstract. | Categories used solely for interpreting results. |

**Supplemental Table 1.** Overview of study characteristics.

| **Article (reference list number)** | **Publication year** | **Intersectionality engagement** | **Region** | **Country ^1^** | **Income quartile ^2^** | **Study design** | **Analytic** | **Unadjusted estimates ^3^** | **Sample characteristics and sample size** | **Social characteristics included** | **Nutrition outcome(s)** |
| --- | --- | --- | --- | --- | --- | --- | --- | --- | --- | --- | --- |
| Abassi et al. (36) | 2019 | Approach | Africa & the Middle East | TUN | LMIC | CS | × | √ | Adults aged 20-49 years in the Greater Tunis region (*n*=2,619). | Gender; geography; family status; education; occupation; economic status/income; age | Dietary intake: (Diet Quality Index-International (DQI-I) >60; and DQI score) |
| Adhikari et al. (37) | 2022 | Framework | South Asia | IND | LMIC | CS | × | × | Children <5 years (*n*=215,554). | Caste; economic status/income; geography; education | Child stunting (height-for-age z-score <-2 sd (moderate), and <-3 sd (severe)); underweight (weight-for-age z-score <-2 sd (moderate), and <-3 sd (severe); and wasting (weight-for-height z-score <-2 sd (moderate) and <-3 sd (severe)) |
| Ailshire et al. (38) | 2011 | Framework | North America | USA | HIC | L | × | √ | White and Black adults aged ≥25 years (*n*=3,426) | Race; gender; economic status/income; education | Body mass index (kg/m²) |
| Alarcao et al. (39) | 2020 | Approach | Europe | PRT | HIC | CS | × | √ | Adults aged 18-84 years (*n*=3,860). | Immigration; gender; age; family status; education; economic status/income | Household food insecurity |
| Assari et al. (41) | 2016 | Framework | North America | USA | HIC | L | √ | × | White and African American older adults aged ≥50 years (*n*=37,495). | Race; gender | Body mass index (kg/m²) |
| Assari et al. (88) | 2017 | Aim | North America | USA | HIC | L | × | √ | White and Black urban youth in 20 U.S cities followed from birth to age 15 (outcome measured at age 15 only) (*n*=1,781) | Race*; gender; education; economic status/income | Body mass index (kg/m²) |
| Assari et al. (42) | 2019 | Aim | North America | USA | HIC | L | √ | √ | White and Black or African American older adults aged ≥50 years (*n*=37,495). | Race and ethnicity*; gender | Body mass index (kg/m²) |
| Atuoye et al. (43) | 2020 | Aim | Africa & The Middle East | TZA | LMIC | CS | √ | × | Adults aged ≥18 years residing in coastal regions Dar es Salaam, Pwani and Tanga (*n*=1,136) | Gender; migration status | Large-scale land acquisition-induced food insecurity |
| Bell et al. (87) | 2019 | Aim | North America | USA | HIC | CS | × | × | Non-Hispanic Black and White men aged ≥20 years (*n*=6,145). | Race; economic status/income; family status | Obesity (BMI ≥30 kg/m²) |
| Bell et al. (90) | 2020 | Framework | North America | USA | HIC | CS | √ | × | Non-Hispanic Black and White adult women (*n*=4,871). | Race; education | Body mass index (kg/m²) |
| Bojorquez et al. (44) | 2015 | Aim | Latin America | MEX | UMIC | CS | × | × | Women aged ≥18 years in Tijuana (*n*=2,268) | Economic status/income; education; immigration and migration status | Dietary intake (consumption patterns determined through factor analysis) |
| Bryn Austin et al. (45) | 2013 | Aim | North America | USA | HIC | CS | × | √ | High school students in four cities (Boston, Massachusetts; Chicago, Illinois; New York City, New York; and San Francisco, California) and 5 states (Delaware, Maine, Massachusetts, Vermont, and Rhode Island) (*n*=24,591) | Ethnicity; sexual orientation; gender | Obesity (BMI>95th percentile for age and gender) |
| Buffarini et al. (46) | 2020 | Framework | Latin America | BRA | UMIC | CS | × | √ | Adolescents aged 15 years from a population-based cohort based in urban Pelotas (*n*=4,101). | Gender; economic status/income | Overweight (BMI for age z-score >+1 sd) |
| Caceres et al. (47) | 2019 | Approach | North America | USA | HIC | CS | × | √ | Sexual minority Black, white and Latina women aged ≥18 years based in the Chicago metropolitan area (*n*=601). | Race and ethnicity*; sexual orientation | Obesity (BMI ≥30 kg/m²) |
| Caceres et al. (48) | 2020 | Framework | North America | USA | HIC | CS | × | × | Adults aged 18-59 years (women: *n*=10,995; men: n=11,310) | Race and ethnicity*; sexual orientation | Body mass index (kg/m²) |
| Chakraborty et al. (49) | 2017 | Framework | South Asia | IND | LMIC | CS | × | √ | Children <5 years (*n*= 46,655) | Economic status/income; caste | Height-for-age percentage of the median |
| Choi et al. (50) | 2020 | Framework | North America | USA | HIC | CS | × | √ | Older adults aged ≥50 years in California (*n=*26,534) | Ethnicity; sexual orientation | Obesity (BMI ≥30 kg/m²) |
| Ciciurkaite (51) | 2021 | Approach | North America | USA | HIC | CS | × | × | Adults aged ≥20 years (*n*=4,640) | Race and ethnicity*; education; gender; economic status/income; | Body mass index (kg/m²) |
| Cohen et al. (52) | 2022 | Framework | North America | USA | HIC | CS | × | × | Black and white adults aged ≥18 years (*n*=359,157) | Race; geography | Obesity (BMI ≥30 kg/m²) |
| Conklin et al. (53) | 2015 | Framework | Europe | GBR | HIC | L | × | √ | Older adults aged >50 years from the population-based EPIC Norfolk Cohort study (*n*=9,580). | Gender; occupation; economic status/income; education; family status | Dietary intake (variety of fruits and vegetables consumed) |
| Cook et al. (54) | 2016 | Aim | North America | USA | HIC | CS | × | × | Asian American adolescents aged 12-17 years in California (*n*=1,533). | Ethnicity; economic status/income | Obesity (BMI>95th percentile for age and gender) |
| Courtney-Long et al. (55) | 2016 | Aim | North America | USA | HIC | CS | × | × | Adults aged ≥18 years with a disability (*n*=473,351) | Race and ethnicity*; education; economic status/income | Obesity (BMI ≥30 kg/m²) |
| Durfee et al. (56) | 2021 | Framework | North America | USA | HIC | CS | √ | × | Employed Black women (with a wage of $11.50 or less) in Raleigh and Minneapolis (*n*=969) | Race; gender; geography | Body mass index (kg/m²) |
| Echeverria et al. (57) | 2017 | Aim | North America | USA | HIC | CS | × | × | U.S.-born non-Latino white adults and Asian American adults aged ≥20 years (*n*=5,484). | Race and ethnicity*; education; immigration status | Obesity (BMI ≥30 kg/m²);  Also explored using BMI ≥25kg/m² for Asian adults. |
| Evans et al. (58) | 2017 | Framework | North America | USA | HIC | CS | × | √ | Non-Hispanic or Latino white, non-Hispanic or Latino Black and Hispanic or Latino adults aged ≥18 years (*n*=32,788). | Race and ethnicity*; economic status/income; education; gender; age. | Body mass index (kg/m²) |
| Evans (59) | 2018 | Framework | North America | USA | HIC | CS | × | √ | Non-Hispanic white, non-Hispanic Black and Hispanic adolescents in grades 7-12 (*n*=13,694). | Race and ethnicity*; immigration status; economic status/income; gender; education; sexual orientation. | Body mass index (kg/m²) |
| Frisco et al. (60) | 2016 | Aim | North America | USA | HIC | CS | × | √ | White children of natives and Mexican-origin youth aged 2-15 years (*n*=10,547). | Ethnicity; gender; immigration status | Obesity (BMI>95th percentile for age and gender) |
| Galobardes et al. (61) | 2000 | Aim | Europe | CHE | HIC | CS | × | √ | Employed adults aged 35-75 years in Geneva (men: *n*=1,767; women: *n*=1,268) | Education; occupation | Body mass index (kg/m²);  Overweight and obesity (BMI ≥25 kg/m²) |
| Gatica-Dominguez et al. (86) | 2019 | Framework | Latin America | GTM | UMIC | CS | × | √ | Children <5 years (*n=*  3,538-12,567) | Indigeneity; geography; economic status/income | Stunting (height-for-age z-score <-2 sd) |
| Goli et al. (62) | 2020 | Aim | South Asia | IND | LMIC | CS | × | × | Rural households in 14 districts across four regions in Uttar Pradesh (*n*=5,087) | Caste; size of agriculture landholding (coded as economic status/income). | Household food insecurity |
| Hargorve (63) | 2018 | Framework | North America | USA | HIC | L | √ | √ | African American or Black adults aged 32-55 years in four US cities: Birmingham, AL; Minneapolis, MN; Chicago, IL; and Oakland, CA (*n*=1,592). | Interviewer-ascribed skin tone (coded as race and/or ethnicity); gender | Body mass index (kg/m²); |
| Hargrove (64) | 2018 | Framework | North America | USA | HIC | L | × | × | U.S born non-Hispanic white, non-Hispanic Black, and Hispanic individuals from adolescence (aged 13) to adulthood (aged 31) (*n*=6,873) | Race and ethnicity*; economic status/income; education; gender | Body mass index (kg/m²) |
| Hernández-Yumar et al. (40) | 2018 | Framework | Europe | ESP | HIC | CS | × | √ | Adults aged ≥18 years (*n*=14,190). | Gender; economic status/income; age; education; family status | Body mass index (kg/m²) |
| Holman et al. (89) | 2020 | Framework | Europe | GBR | HIC | CS | × | √ | Older adults aged ≥50 years (*n*=16,437). | Gender; economic status/income; ethnicity; education | Body mass index (kg/m²) |
| Kanchi et al. (65) | 2018 | Framework | North America | USA | HIC | CS | × | √ | New York City residents aged ≥20 years (*n*=1,527). | Race and ethnicity*; gender | Overweight and obesity (BMI ≥25 kg/m²) |
| Kochupurackal et al. (66) | 2021 | Approach | South Asia | IND | LMIC | CS | × | × | Children <5 years (*n*=206,276). | Caste; gender; economic status/income; geography | Composite index of anthropometric failure |
| Leroy et al. (67) | 2014 | Aim | Latin America | MEX | UMIC | CS | × | × | Mothers (12-49 years) with a child (<5 years) in 228 rural communities in the eight poorest state of the country  (*n*=1,547) | Economic status/income; education | The double burden of malnutrition: child stunting (height-for-age z-score <-2 sd) with overweight mother (BMI ≥25 kg/m²) |
| Liew (68) | 2020 | Framework | North America | USA | HIC | L | × | √ | Older adults aged >50 years (sample sizes not reported). | Race and ethnicity*; gender; education | Body mass index (kg/m²) |
| McClain et al. (69) | 2021 | Framework | North America | USA | HIC | CS | √ | √ | Younger adults aged 24-32 years *(n=*4,847) | Race and ethnicity*; gender | Body mass index (kg/m²)  Waist circumference (cm) |
| Mukhopadhyay et al. (70) | 2018 | Framework | South Asia | IND | LMIC | CS | × | × | Children <5 years (*n=*46,655). | Relgion; gender; caste | Stunting (height-for-age z-score <-2 sd);  Height-for-age percentage of the median |
| Mukhopadhyay (71) | 2015 | Framework | South Asia | IND | LMIC | CS | × | × | Children <5 years in rural areas of India (*n=*46,655). | Caste; gender; economic status/income | Stunting (height-for-age z-score <-2 sd) |
| Mukhopadhyay (72) | 2015 | Framework | South Asia | IND | LMIC | CS | × | × | Children <5 years (*n=*46,655). | Caste; gender; economic status/income | Stunting (height-for-age z-score <-2 sd) |
| Myers et al. (73) | 2017 | Aim | North America | USA | HIC | CS | × | √ | Non-Latino white, non-Latino Black and Latino adults aged ≥18 years (*n*=32,464) | Race and ethnicity*; immigration status | Food insecurity |
| Newby et al. (74) | 2012 | Aim | North America | USA | HIC | CS | × | × | Black and white older women aged ≥45 years (*n*=12,105). | Race; geography | Nutrient intake |
| Patterson et al. (75) | 2020 | Framework | North America | USA | HIC | CS | × | × | Black and white women aged 18-59 years (NHIS data: *n*=47,596; NHANES data: *n*=5,106) | Race; sexual oritentation | Household food insecurity (past 12 months and past 30 days) |
| Perreira et al. (76) | 2018 | Framework | North America | USA | HIC | L | √ | √ | White, Black, Asian and Hispanic adolescents (aged 12-18 years) through to adulthood (ages 24-32 years) (*n*=11,470).  Note: outcome assessed in adulthood only. | Race (interviewer-ascribed skin tone); gender | Obesity (BMI ≥30 kg/m²); Body mass index (kg/m²) |
| Polos et al. (77) | 2021 | Framework | North America | USA | HIC | L ^4^ | √ | × ^a^ | Black and non-Black adolescents (aged 12-18 years) through to adulthood (ages 33-40)  (*n*=5,274-5,482 - sample size fluctuates between survey waves) | Race; gender; economic status/income | Obesity (BMI ≥30 kg/m² or BMI >95^th^ percentile for age and gender) |
| Prus et al. (78) | 2010 | Aim | North America | USA; CAN | HIC | CS | × | × | White and non-white adults aged ≥45 years (*n*=8,688). | Race; immigration status; geography | Obesity (BMI ≥30 kg/m²) |
| Riley et al. (79) | 2018 | Approach | Africa & The Middle East; East Asia | MOZ; CHN | LIC; UMIC | CS | × | × | Households in Maputo (MOZ) (*n*=1,957) and Nanjing (CHN) (*n*=1,081) | Gender (of household head); age; family status; geography; education. | Household food insecurity |
| Rodriguez-Alvarez et al. (80) | 2018 | Aim | Europe | ESP | HIC | CS | × | √ | Adults between ages 18-64 years (*n*=27,720). | Gender; education; immigration status | Obesity (BMI ≥30 kg/m² |
| Salsberry et al. (81) | 2007 | Aim | North America | USA | HIC | CS | × | √ | Non-Hispanic white, non-Hispanic Black, and Mexican American adults (*n*=3,049). | Gender; economic status/income; race and/or ethnicity | Abdominal obesity (waist circumference > 102cm for men and >88 cm for women) |
| Tichenor et al. (82) | 2015 | Aim | North America | USA | HIC | CS | × | × | Adults aged ≥18 years (*n*=275,864). | Race and ethnicity*; geography | Dietary intake (fruit and vegetable consumption at least once per week) |
| Waldstein et al. (83) | 2016 | Aim | North America | USA | HIC | CS | × | × | Black or African American and white or Caucasian adults aged 30-64 years in thirteen neighbourhoods in Baltimore, Maryland (*n*=2,270). | Race; economic status/income; gender | Body mass index (kg/m²)  Waist circumference (cm) |
| Walsemann et al. (85) | 2011 | Aim | North America | USA | HIC | L | × | × | White, Black or Hispanic adolescents and young adults (aged 14-21 years) through to adulthood (aged 43-52 years) (*n*=8,354) | Race and ethnicity*; education; gender | Body mass index (kg/m²) |
| Walsemann et al. (84) | 2017 | Aim | North America | USA | HIC | CS | × | × | Latino, Asian or U.S.-born white adults aged ≥18 years in California state (*n*=245,679). | Ethnicity and immigration status. | Household food security |

Note: CS=cross-sectional; L=Longitudinal.^1^ ISO ALPHA-3 abbreviation; ^2^ World Bank classification - Gross National Income per capita. ^3^ Unadjusted estimates include studies that provided unadjusted estimates even if accompanied by adjusted results; results are considered unadjusted when not adjusted for sociodemographic factors that are not part of the intersectional social characteristics of interest with the exception of age; ^4^ Longitudinal, quasi-experimental design. * Authors refer to “race/ethnicity”, or race and ethnicity are used interchangeably.

**Supplemental Table 2.** Intersectional relationships between social characteristics investigated in included articles with reference list number (data underlying Figure 3).

| Race and/or ethnicity |  |  |  |  |  |  |  |  |  |  |  |  |  |
| --- | --- | --- | --- | --- | --- | --- | --- | --- | --- | --- | --- | --- | --- |
| Gender | 38, 41, 42, 45, 51, 56, 58, 59, 60, 63, 64, 89, 65, 68, 69, 76, 77, 81, 83, 85 |  |  |  |  |  |  |  |  |  |  |  |  |
| Economic status/income | 38, 88, 87, 51, 54, 55, 58, 59, 64, 89, 77, 83 | 36, 38, 88, 46, 51, 53, 58, 59, 64, 40, 89, 66, 71, 72, 77, 81, 83 |  |  |  |  |  |  |  |  |  |  |  |
| Education | 38, 88, 90, 51, 55, 57, 58, 59, 64, 89, 68, 85 | 36, 38, 88, 51, 53, 58, 59, 64, 68, 40, 89, 79, 80, 85 | 44, 58, 59, 40, 89, 67 |  |  |  |  |  |  |  |  |  |  |
| Geography | 52, 56, 74, 78, 82 | 36, 56, 66, 79 | 66 | 79 |  |  |  |  |  |  |  |  |  |
| Immigration or migration status | 57, 59, 60, 73, 78, 84 | 39, 43, 59, 60, 80 | 39, 44, 59 | 39, 44, 57, 59, 80 | 78 |  |  |  |  |  |  |  |  |
| Caste | - | 66, 70, 71, 72 | 37, 49, 62, 66, 71, 72 | 37 | 37, 66 | - |  |  |  |  |  |  |  |
| Age | 58 | 36, 58, 40, 79 | 58, 40 | 58, 40 | 79 | 39 | - |  |  |  |  |  |  |
| Sexual orientation | 45, 47, 48, 50, 59, 75 | 45, 59 | 59 | 59 | - | 59 | - | - |  |  |  |  |  |
| Family status | 87 | 36, 53, 40, 79 | 87, 40 | 40 | 79 | 39 | - | 40 | - |  |  |  |  |
| Occupation | - | 36, 53 | - | 61 | - | - | - | - | - | - |  |  |  |
| Religion | - | 70 | - | - | - | - | - | - | - | - | - |  |  |
| Indigeneity | - | - | 86 | - | 86 | - | - | - | - | - | - | - |  |
|  | Race and/or ethnicity | Gender | Economic status/income | Education | Geography | Immigration or migration status | Caste | Age | Sexual orientation | Family status | Occupation | Religion | Indigeneity |

**Supplemental Table 3**. List of data sources used in included articles

| **Data source** | **Reference list number of included article** |
| --- | --- |
| National Longitudinal Study of Adolescent to Adult Health (Add Health) ^a^ | 59, 69, 76, 77 |
| Americans’ Changing Lives Survey ^a^ | 38 |
| Baseline information from Minneapolis and Raleigh, North Carolina, that reflects wages before the implementation of the Minneapolis minimum wage ordinance | 56 |
| Behavioral Risk Factor Surveillance System | 52, 55, 82. |
| Bus Sante´ 2000 | 61 |
| California Health Interview Survey | 50, 54, 84 |
| Coronary Artery Risk Development in Young Adults (CARDIA) Study | 63 |
| Cross-sectional study conducted in Dar es Salaam, Pwani and Tanga of Tanzania | 43 |
| Data from the “Obe-Maghreb” research project | 36 |
| EPIC Norfolk cohort | 53 |
| European Health Survey in Spain (EHSS) | 80 |
| Fragile Families and Child Wellbeing Study | 88 |
| Giri Institute for Development Studies (GIDS) in 2014–15 in 14 districts across four regions in the state of UP under the project “Social and Educational Status of OBC/Dalit Muslims in Uttar Pradesh” | 62 |
| Guatemala Demographic Health Surveys | 86 |
| HCP household food security surveys (Mozambique & China) | 79 |
| Healthy Aging in Neighborhoods of Diversity across the Lifespan | 83 |
| India National Family Health Survey (DHS) | 37, 49, 66, 70, 71, 72 |
| National Health and Nutrition Examination Survey (NHANES) ^a^ | 87, 90, 48, 51, 57, 60, 73, 81, 75 |
| National Health Interview Survey (NHIS) | 75 |
| National Longitudinal Survey of Youth ^a^ | 64, 85 |
| New York City Health and Nutrition Examination Survey (NYC NHANES) | 65 |
| Population-based cohort study in Pelotas, a Southern Brazilian city | 46 |
| REGARDS | 74 |
| Representative survey of adult women in Tijuana. | 44 |
| Rural communities selected randomly from the pool of eligible communities in 8 of the poorest states in the country (Veracruz, Chiapas, Tabasco, Guerrero, Oaxaca, Quintana Roo, Campeche, and Yucatan). | 67 |
| Spanish National Health Survey (SNHS) | 40, 80 |
| The Chicago Health and Life Experience of Women (CHLEW) study ^b^ | 47 |
| The English Longitudinal Study of Ageing (ELSA) & Understanding Society: the United Kingdom Household Longitudinal Study (UKHLS) | 89 |
| The Health and Retirement Study | 41, 42, 68 |
| The Joint Canada/United States Survey of Health (JCUSH) | 78 |
| The National Epidemiologic Survey on Alcohol and Related Conditions (NESARC) ^a^ | 58 |
| The National Food, Nutrition and Physical Activity Survey | 39 |
| Youth Risk Behaviour Surveillance Systems (YRBSS) | 45 |

Note: ^a^ Oversampling of groups with certain social characteristics noted by at least one author using the data source;  ^b^ convenience sample and a supplemental sample of groups with certain social characteristics.

**Supplemental Table 4.** Statistical methods used for descriptive approaches to intersectionality among included articles.

| **Method** | **Reference list number of included article** |
| --- | --- |
| Regression using a single variable representing intersectional groups | 43, 47, 48, 57, 60, 65, 78, 84, 73, 66, 38 |
| Regression using multiple variables representing intersectional groups | 70, 71, 72 |
| Stratified regression only ^1^ | 55, 50, 81, 63 |
| Regression with interactions between social characteristics | 88, 64, 45, 51, 54, 59, 65, 67, 68, 75, 82, 85, 76, 74, 83, 52, 36, 46, 44, 53, 39, 61, 80, 37, 87, 62 |
| Interaction tested on… |  |
| Additive-scale from linear regression | 88, 64, 51, 59, 65, 67, 68, 85, 76, 74, 36, 44, 53, 61 |
| Additive scale only from non-linear (e.g., logistic, Poisson) regression | 75 |
| Multiplicative scale only from non-linear (e.g., logistic, Poisson) regression | 45, 54, 82, 76, 52, 42, 46, 39, 61, 80, 87 |
| Additive and multiplicative scale from non-linear (e.g., logistic, Poisson) regression | 65 |
| Interaction not reported but instead, predictive probabilities estimation from non-linear (e.g., logistic, Poisson) regression | 37, 62 |
| Growth curve modelling in a multi-level framework | 63, 64, 68, 85, 38 |
| Multi-level analysis of individual heterogeneity and discriminatory accuracy (MAIHDA) | 58, 59, 40, 89 |
| Decomposition analysis: Correct Method of General Entropy Class Index | 70, 49 |
| Descriptive analysis only | 79, 86 |

Note: Methods used are not mutually exclusive to articles. ^1^ Some studies included Chow tests for equality of coefficients between stratified regression models. Measures for additive scale interaction from non-linear regression models included relative excess risk due to interaction (RERI) (65,75), attributable proportion (75), synergy index (75), and the ratio of observed to expected joint effects on the relative scale (75).

**Supplemental Results 1. Narrative synthesis of included articles with similarities at the regional level.**

Of the five studies based in Europe, two used a multi-level analysis of individual heterogeneity and discriminatory accuracy (MAIHDA) and investigated intersections including gender, wealth and education (the study in Spain also included age and family status, and the study in the U.K. additionally included race and/or ethnicity); both found no evidence of ‘intersectionality effects’, as most of the variance in BMI was explained by the additive main effects of the social characteristics (40,89). Two studies investigated gender by immigration status; there was no evidence that gender and immigration status interacted on food insecurity in Portugal (42), whilst in Spain, evidence showed that native men were more likely to be obese than foreign-born men, whilst the opposite occurred among women (80).

Studies in Latin America were considerably diverse, but three of four investigated anthropometric outcomes. Evidence from Brazil showed that higher parental income was associated with a higher risk of obesity among adolescent boys but not girls (46), whilst evidence from Mexico showed how greater household wealth was associated with a higher prevalence of stunted child-overweight mother pairs but only in households where the mother had not completed primary educated (60). The third explored the intersectionality of indigeneity by wealth and rurality on stunting prevalence among children under five years in Guatemala and how these trends changed between 1995 and 2014 (86) and showed how, in 2014, poor and rural indigenous children were twenty years behind non-indigenous children with similar characteristics in terms of stunting prevalence.

As in Latin America, the three studies in Africa & the Middle East were also diverse, but all focused on gender: in a coastal region of Tanzania, large-scale land acquisition-induced food insecurity was shown to vary across intersections of gender and migration status, with male non-migrant, and male and female migrants reporting greater food insecurity than female non-migrants (43). In Tunisia, gender was intersected separately with seven social characteristics: compared with women, men consistently had a greater average diet quality index-international (DQI) score and probability of a DQI score>60 besides in rural areas, between ages 40-49 years, among those with no primary school, and those that are retired/not working (36). The last study in Africa & the Middle East, based in Maputo, Mozambique, also included populations in Nanjing, China (East Asia), which showed how ‘female-headed household’ is a more useful characteristic for understanding gender-food insecurity linkages in each city when intersected with other household characteristics (79). For example, among female-headed households, the youngest female heads were the least food insecure in Maputo but the most food insecure in Nanjing, and among those that are unmarried/unpartnered, male household heads were less food insecure than female household heads in Nanjing, but female-households heads were less food insecure than male in Maputo.
